# Bioengineered Chitosan–Collagen–Honey Sponges: Physicochemical, Antibacterial, and In Vitro Healing Properties for Enhanced Wound Healing and Infection Control

**DOI:** 10.3390/polym17172379

**Published:** 2025-08-31

**Authors:** David Servín de la Mora-López, Leticia Olivera-Castillo, Jaime López-Cervantes, Dalia I. Sánchez-Machado, Jesús Fernando Ayala-Zavala, Herlinda Soto-Valdez, Tomás J. Madera-Santana

**Affiliations:** 1 Coordinación de Tecnología de Alimentos de Origen Vegetal, Centro de Investigación en Alimentación y Desarrollo, A.C. Carretera Gustavo Enrique Astiazarán Rosas No. 46. Colonia La Victoria, Hermosillo 83304, Sonora, Mexico; davidserml@hotmail.com (D.S.d.l.M.-L.); jayala@ciad.mx (J.F.A.-Z.); hsoto@ciad.mx (H.S.-V.); 2 Departamento Recursos del Mar, Centro de Investigación y de Estudios Avanzados del IPN-Unidad Mérida, Carr. Ant. a Progreso Km. 6, Mérida 97310, Yucatán, Mexico; loliveracastillo@gmail.com; 3 Departamento de Biotecnología y Ciencia de los Alimentos, Instituto Tecnológico de Sonora, Calle Chihuahua, Centro, Urb, No. 1. Colonia Centro, Ciudad Obregón 85000, Sonora, Mexico; dsanchez@itson.edu.mx

**Keywords:** chronic ulcers, bacterial infection, sponge dressings, honey, chitosan, collagen

## Abstract

Bacterial-mediated infections represent a major risk factor for chronic wounds. Numerous polymeric dressings have been proposed to reduce this incidence and promote wound healing. In the present investigation, chitosan/collagen/honey-based sponges were prepared by freeze-drying. The effect of honey incorporation at different concentrations on the physicochemical and antibacterial properties of the sponges was evaluated. The SEM images showed that the surface and cross-sections of all samples had a porous structure. The pore size gradually increased in the range of 78.14 to 126.9 μm due to the increase in honey content in the sponges. This property resulted in considerably higher porosity degrees (79.90–90.13%) and absorption rates (ranges of 1357–1665% in deionized water and 865–1938% in PBS solution) in honey-loaded systems. Conversely, the honey composite formulations exhibited a reduction in permeability, with WVTR values ranging from 131.01 to 99.39 gh^−1^m^−2^ and values of WVP from 0.3255 to 0.2118 gm^−1^d^−1^mm Hg^−1^. The mechanical properties showed that adding honey made the sponges more flexible (12.49–7.95% MPa) but decreased elongation rates in the sponges (16.36–7.56%) due to higher pore heterogeneity. The antibacterial tests indicated that all treatments had inhibitory effects against *S. aureus*, *P. aeruginosa*, *E. coli*, and *L. monocytogenes*. The results from cells viability assays and in vitro healing models using human keratinocytes demonstrate that chitosan/collagen/honey sponges represent a potential alternative for applications such as wound dressings to help treat skin ulcers. The physicochemical, antibacterial, and biocompatibility properties of chitosan/collagen/honey sponges indicated their potential as a promising alternative for clinical use.

## 1. Introduction

Chronic wounds have a strong socioeconomic impact worldwide. In the United States of America, chronic wounds have affected more than 6.5 million patients, and the health care expenditure exceeds USD 25 billion annually for the treatment of wound-related complications [1].

Usually, the wound healing process consists of four overlapping stages: hemostasis, inflammation, proliferation, and cell maturation [2]. However, bacterial-mediated infections disrupt these processes in chronic wounds, as they can keep the inflammatory phase active for a prolonged period [3]. According to reports, 80–90% of mild infections are caused by *Staphylococcus aureus*, whereas *Enterococcus* and *Pseudomonas* spp. cause moderate illnesses [4]. In chronic wounds, the massive invasion of these microorganisms causes a decrease in the nutrient and oxygen supply from the bloodstream, triggering severe complications such as limited joint mobility, tissue necrosis, or tissue amputation [4,5]. Numerous antibiotics have been introduced to reduce the incidence. However, extended treatment durations have led to species originating from clinical settings becoming more likely to evolve new resistance mechanisms against antibiotics [6]. Researchers have proposed using affordable natural materials as a potential option to eliminate infections and assist wound healing.

Chitosan is a natural amino saccharide derived from the N-deacetylation of chitin [7]. Chitosan is a biodegradable and biocompatible copolymer consisting of N-acetyl-D-glucosamine and D-glucosamine molecules linked by β-(1,4)-bonds [8]. The free amino groups give chitosan natural antibacterial properties against bacteria like *Escherichia coli*, *Salmonella typhi*, *Staphylococcus aureus*, and *Listeria* spp. [4,9]. Some studies have reported that chitosan promotes granulation, accelerates inflammatory cell infiltration, and stimulates the migration and proliferation of fibroblasts in wound healing [7,10,11]. Thus, chitosan has been used to treat various skin ulcers [12]. However, chitosan’s main disadvantage is its poor mechanical strength, which has greatly limited its application in biomedicine. The alternative to this drawback is reinforcing its polymeric structure through other polymers or plasticizing agents [9,10].

Collagen is the main extracellular matrix structural component (ECM) and the predominant protein of skin, bones, tendons, and other connective tissues [13,14,15]. Collagen is an important biopolymer in biomedicine due to its biodegradable, biocompatible properties and low antigenicity [15,16]. According to reports, collagen has several biologically active locations where its cellular adhesive peptides and receptors can improve the body’s reactions to processes like cell adhesion, migration, proliferation, and differentiation, encouraging the formation of new tissues [2,17]. Several studies have shown chitosan/collagen-based biomaterials significantly improve healing responses because they have physical, chemical, and biological properties analogous to the dermis [16].

Honey has been widely used as an efficient remedy for skin ulcer treatment [6]. It is well-documented that the healing effect of honey is due to its antimicrobial and anti-inflammatory properties [18,19]. Also, honey favors autolytic debridement, reduces pain and inflammation, provides moist environments, stimulates fibroblast proliferation, and promotes angiogenesis [20,21]. The antioxidant capacity conferred by flavonoids and phenolic acids presents anti-inflammatory properties in wounds [22]. Similarly, honey’s high osmotic effect, acidic nature, constant hydrogen peroxide production, and phytochemical compounds determine its antimicrobial activity [21]. Honey has been shown to exhibit strong inhibition against microorganisms isolated from infected chronic wounds, and these have included *S. aureus*, *S. epidermidis*, *P. aeruginosa*, *Klebsiella pneumoniae*, *E. coli*, *E. faecium*, and *Candida albicans* [6,18]. Recently, the incorporation of honey into different kinds of polymeric dressings for wound healing applications has been documented. However, the concept of incorporating honey into chitosan–collagen-based matrices has not been thoroughly studied. Therefore, our hypothesis suggests that the inclusion of honey in chitosan–collagen sponges could provide and enhance beneficial effects in wound healing.

According to the positive effects of chitosan, collagen, and honey on wound healing, the mixture of these components could lead to the formation of a dressing with improved physicochemical, antibacterial, and regenerative characteristics. Therefore, we focused the present work on investigating the properties and benefits of combining these components as wound dressings.

## 2. Materials and Methods

### 2.1. Materials

The chitosan used had a deacetylation degree of 87% and a molecular weight of 378 kDa; it was provided by QUITOMEX, S.A. (Obregón, Mexico). Hydrolyzed collagen from fish scales was supplied by the Food Science and Technology Laboratory of the Instituto Tecnológico de Sonora (Obregón, Mexico). *Apis mellifera* honey was acquired from a local market (Maní, Mexico). Glacial acetic acid was purchased at FAGALAB (Mocorito, Mexico). Phosphate-buffered saline (PBS) solution was supplied by Sigma-Aldrich (KgaA, Darmstadt, Germany). All chemicals were reagent grade.

### 2.2. Preparation of Chitosan–Collagen–Honey Sponges

Chitosan, collagen, and honey composite sponges were prepared according to the freeze-drying method proposed by Martinez-Ibarra et al. [9], with some modifications. Previously, a chitosan (2% *w*/*v*) and collagen (0.25% *w*/*v*) blend was prepared in acetic acid (1% *v*/*v*). This formulation was referred to as ChC. In addition, a pure chitosan solution (2% *w*/*v*) was separately prepared and considered a sample control (Ch 2%). Through the ChC blend, three other formulations were prepared by incorporating honey at concentrations of 20%, 40%, and 60% w based on the total mass of polymers in solution, and these formulations were referred to as ChC-20H, ChC-40H, and ChC-60H, respectively. The concentrations mentioned above were selected based on preliminary studies conducted previously. Table 1 shows the composition of the prepared sponges. The mixtures were placed under constant magnetic stirring for 3 h until their components were completely integrated, and subsequently, the blends were filtered on organza cloth. Then, 12 mL of the resulting mixtures was transferred to plastic molds (85 mm diameter), and these were subjected to freeze-drying in a lyophilizer (LyoBeta, Telstar, Spain) for 20 h. The freezing process was performed at −40 °C under a vacuum pressure of 200 μbar, and the freeze-drying was carried out at an initial temperature of −40 °C that was increased until reaching 20 °C under a controlled ramp rate of 1 °C/min. Finally, the sponges produced were extracted from the plastic molds and stored in LDPE airtight bags at 25 ± 2 °C of temperature for protection against humidity until further characterization.

### 2.3. Physicochemical Characterization of Sponges

#### 2.3.1. Color Measurement

Color properties of the chitosan–collagen–honey sponges were determined using a colorimeter (Chroma Meter CR-400, Konica Minolta, Osaka, Japan) calibrated with a standard (Y = 94.1, X = 0.3129, and y = 0.3191). Using the CIE Lab system, the color parameters measured were L* (lightness), a* (red-green coordinates), and b* (yellow-blue coordinates) in three specific regions of the sponge, and we reported the average of 9 measurements for each sample. We determined color difference (Δ*E*) to evaluate the color changes caused by incorporating the compounds into the pure chitosan sponges (Ch). Thus, we calculated Δ*E* using the following Equation (1):

(1) $$\Delta E=\sqrt{{\left(∆L*\right)}^{2}+{\left(∆a*\right)}^{2}+{\left(∆b*\right)}^{2}}$$

where ∆L*= *L* − *L_o_*; ∆a* = *a* − *a_o_*; and ∆b* = *b* − *b_o_*. The L*, a*, and b* values are the chromatic parameters of the sample, while L_o_*, a_o_*, and b_o_* correspond to the color parameters of Ch 2% sponges.

#### 2.3.2. Fourier Transform Infrared Spectroscopy (FTIR)

The chemical–structural characterization of chitosan–collagen–honey sponges was determined through FTIR spectra using an Attenuated Total Reflectance (ATR) accessory coupled to a Nicolet iS50 FTIR spectroscope (Thermo Fisher Scientific, Waltham, MA, USA). For each sponge treatment, 32 scans with wavenumbers ranging between 4000 and 600 cm^−1^ and a spectral resolution of 4 cm^−1^ were performed.

#### 2.3.3. Morphological Analysis

The surface and cross-sectional morphology of the sponges were analyzed using a Field Emission Scanning Electron Microscope (FE-SEM) JEOL JSM-7600F (Peabody, MA, USA). Previously, the samples were fixed with double-sided carbon adhesive tape in aluminum tubular cells. Subsequently, they were coated with a thin Au/Pd composite layer using a Quorum model QI5OR-EN sputtering instrument (Sussex, UK). The micrographs were obtained under scales of 200 μm and magnifications of 150× in the surface section and 200× in the cross-sectional region. Moreover, the pore size of the sponges was determined through the micrographs taken using ImageJ software (ver. 1.54i 3 March 2024). The pore size was obtained by taking the micrograph scale as a reference. Pore size distribution plots and the mean pore size were obtained based on 50 measurements per treatment.

#### 2.3.4. Porosity Degree

Porosity degree of the sponges was studied using a method called liquid displacement, which was proposed by Chandika et al. [23], with some modifications. Before this study, the sample dimensions were measured to calculate their volume. A known weight (*W_o_*) sample was immersed in ethanol solution (3 mL) due to its low surface tension and kept in adsorption equilibrium for 10 min. Then, the remaining moisture was removed from the dressing surface using filter paper. The wet sample weight (*W_f_*) was recorded, and the porosity degree was calculated using Equation (2):

(2) $$Porosity\;(\%)=\frac{W_{f}-W_{o}}{\rho \;V_{s}}$$

where *W_f_* corresponds to the sponge weight after remaining in absorption equilibrium for 10 min in ethanol; *W_o_* is the initial sponge weight; *ρ* is the 96% ethanol density; and vs is the sponge volume.

#### 2.3.5. Absorption Capacity

The absorption capacity of the chitosan–collagen–honey sponges was tested according to the methodology proposed by Valenzuela-Rojo et al. [24], with some modifications. The assays were performed using deionized water and PBS (pH = 7.4) as hydrating solutions. Thus, a sample of known weight (*W_o_*) was kept in contact with 3 mL of the hydrating solution, and its weight was monitored under different time intervals in absorption equilibrium (5, 10, 15, 30, 45, and 60 min). To perform the weight monitoring, the sponges were removed from the hydrating solution and wiped dry using filter paper to eliminate excess water. Then, the wet sponge weight was recorded (*W_f_*), and the absorption capacity or percentage of swelling (%*SW*) was determined from Equation (3):

(3) $$\%SW=\frac{W_{f}-W_{o}}{W_{o}}\;\times \;100$$

where *W_o_* represents the initial sponge weight and *Wf* is the wet sponge weight that remained in adsorption equilibrium with PBS or deionized water at a given time interval. The absorption capacity was determined according to the mean value of four replicates per treatment.

#### 2.3.6. Barrier Properties

The tests for the determination of water vapor transmission rate (WVTR) and water vapor permeability (WVP) of the sponges were performed according to the wet cup gravimetric method described by ASTM E96 [25]. The water vapor transport of the sponges was determined by the progressive weight loss of a transmission container (*w*). Thus, a sponge sample was firmly fixed on top of a transmission container containing 30 mL of deionized water. To maintain a stable temperature and relative humidity during the assay, the container with the sample was stored in a desiccator containing dry silica. Subsequently, the container weight was periodically recorded for 30 h, and the measurements were performed in triplicate for each treatment. To calculate WVTR, the container weight difference (g) vs. time (h) was plotted. From the generated slope, it was followed by Equation (4):

(4) $$WVTR=\frac{w}{t\times A}$$

where *w* represents the container weight loss (g); *t* indicates the time in hours; and *A* is the permeation area (1.54 × 10^−4^ m^2^). Likewise, WVP was obtained from WVTR values, and following Equation (5):

(5) $$WVP=\frac{WVTR\times l}{\Delta p}$$

where *l* is the sponge thickness and Δ*p* represents the difference between internal and external water vapor pressures of the container where the sample was fixed.

#### 2.3.7. Mechanical Properties

The mechanical properties of the chitosan–collagen–honey sponges were tested using a TA-XT plus Stable Micro-Systems (Surrey, UK), following the procedures set by the ASTM D882-02 standard method [26]. The parameters to be evaluated were tensile strength, elongation at break, and elasticity modulus. Initially, test specimens (10 mm × 60 mm) were obtained from the samples, and the thickness of each one was measured in triplicate. The distance between clamps was 30 mm. The tests were performed at a crosshead speed of 0.1 mm/s until failure was reached. Through the stress–strain curves obtained, the mechanical parameters were calculated according to the mean value of six measurements per treatment.

#### 2.3.8. Thermal Properties

The thermal properties of the sponges were measured using thermogravimetric analysis (TGA) and differential scanning calorimetry (DSC). To evaluate the thermal stability of the sponges by TGA, thermograms of TGA/DTGA were obtained from a TGA Discovery from TA Instruments Inc. (New Castle, DE, USA). A sample weight of 6–8 mg was loaded into a platinum cell. Then, the cell was placed in the thermobalance and heated from 30 °C to 700 °C at a heating rate of 10 °C/min under a constant nitrogen flow of 50 mL/min.

The DSC analysis was performed using a DSC Discovery from TA Instruments Inc. (New Castle, DE, USA). A sponge weight of 1–5 mg was loaded into a hermetic aluminum cell that was subsequently sealed under pressure using a Tzero Press from TA Instruments Inc. (New Castle, DE, USA), and an empty cell was used as a reference. The cells were heated from −10 °C to 180 °C at a heating rate of 10 °C/min, and after heating, the samples were cooled to 25 °C. Finally, the endothermic transition parameters, maximum dehydration temperature (T*_D_*) and endothermic enthalpy (Δ*H*), were determined from the DSC thermograms obtained.

### 2.4. Antibacterial Activity

The antibacterial effect of the chitosan–collagen–honey sponges was evaluated following the microplate turbidimetric growth inhibition method proposed by Gonzalez-Perez et al. [27], with some modifications. Gram-positive strains, including *S. aureus* (ATCC 6538) and *L. monocytogenes* (ATCC 7644), and Gram-negative strains, such as *P. aeruginosa* (ATCC 10154) and *E. coli* (O157:H7 K3999), were used for the analysis, which represent the main microorganisms with high prevalence involved in chronic infections.

Previously, a bacteria loop was inoculated into tubes containing 10 mL of Oxoid™ Brain and Heart Infusion broth (BHI: composition of 12.5 g/L brain infusion solids, 5.0 g/mL beef heart infusion solids, 10.0 g/L proteose peptone, 2.0 g/L glucose, 5.0 g/L sodium chloride, and 2.5 g/L disodium phosphate) (Thermo Fisher Scientific, Loughborough, UK) and incubated at 37 °C for 18 h. Then, the inoculum concentration was adjusted to 1 × 10^6^ CFU/mL using saline solution (NaCl 0.9% *w*/*v*) as a diluting agent.

Samples of each treatment (5 mm diameter discs) were prepared and kept under UV irradiation for 30 min/side to ensure sterility. To perform the analysis, 5 μL of the inoculum was added to each well of a 96-well microplate and supplemented with BHI broth until a final volume of 300 μL. Subsequently, sponge samples were deposited in the microplate wells. For comparison, wells containing the inoculum without sponges were used as controls. Also, to verify the assay’s sterility, a triplicate of wells with sterile BHI broth without treatments and another triplicate of wells with sterile BHI broth containing the treatments were prepared. The microplate was transferred to a FLUOStar Omega microplate reader (BMG LabTech, Ortenberg, Germany) with incubation at 37 °C for 18 h. Optical density (OD) readings at 625 nm every 30 min and shaking for 10 s before each reading were performed. Finally, the antibacterial activity of the sponges was reported by plotting the OD readings vs. time (h) using OriginPro 2016 software from OriginLab Corp. (Northampton, MA, USA). The data were presented based on the mean value of three replicates.

### 2.5. Cell Viability Tests

The cytotoxicity and cell viability assays were performed according to the methodology proposed by Olivera-Castillo, which is described as follows. Studies were performed with the skin cells that are mainly involved in the healing process, mainly human immortalized keratinocytes (HaCaTs). The same HaCaT cells utilized in the study by Servín de la Mora et al. [28] were employed for the analysis. This study considered only Ch 2%, ChC, and ChC-40H treatments because they presented the best cell viability results according to preliminary studies.

HaCaTs were prepared according to the established methodology by Olivera-Castillo et al. [29], by culturing the HaCaTs in 75 cm^2^ slant-neck cell culture flasks (Nest, Biotech, Jiangsu, China) containing Roswell Park Memorial Institute (RPMI) medium enriched with 10% fetal bovine serum (10% FBS). Cells were incubated in a humidified atmosphere at 37 °C with 5% CO_2_ until they reached a confluence of 80%. The RPMI medium with 10% FBS was replaced every 48 h with pre-washing of the cells using PBS solution. Once 80% cell confluence was reached, the RPMI medium was removed from the flasks, and then the cells were detached with 2 mL of trypsin + 0.05% EDTA solution. The cell culture flasks were incubated with trypsin for 5 min at 37 °C, and subsequently, 4 mL of RPMI medium (10% FBS) was added to inactivate the enzymatic activity. Cell suspensions were recovered in Eppendorf tubes and centrifuged at 1200 RPM for 5 min. Subsequently, the supernatant was discarded, and the cell pellet was homogenized with a fresh RPMI medium. Then, a cell count was performed.

#### 2.5.1. Effects of the Sponges on Cell Viability In Vitro

In order to perform the viability assay in the treatments, samples of five mm diameter sponges (spunches) were previously irradiated with UV for 30 min/side and subsequently soaked with RPMI medium. The sponges were transferred to 48-well microplates, where 5 × 10^4^ HaCaT/well were later seeded. The microplate was incubated at 37 °C in a humidified atmosphere with 5% CO_2_. After 24 h, cell growth was observed and analyzed in the peripheral and inner regions of the sponges using an optical microscope.

#### 2.5.2. In Vitro Cellular Healing Test

In vitro cell healing studies were performed according to the methodology proposed by Monsuur et al. [30], with some modifications. Previously, sponge samples that were treated with irradiation for 30 min on each side were soaked in RPMI medium (10% FBS) and then placed in a 48-well microplate that had about 5 × 10^4^ HaCaT/well. The microplate was incubated under humidified conditions (37 °C and 5% CO_2_), and after 24 h, the sponge samples were removed from the microplate wells. This action favored the treatments to form a hole (wound) in the center of the cell suspension due to the absorption of cells produced by the samples during the incubation process. Then, the microplate was incubated again, and the cell activity in the wound was constantly monitored by optical microscopy.

### 2.6. Statistical Analysis

All quantitative data were presented according to mean values ± standard deviations. The color, mechanical, transport (barrier), porosity degree, and pore size properties were evaluated through a one-way ANOVA design using a Tukey’s test with a significance level of *p* < 0.05. The antimicrobial activity evaluated through the microplate turbidimetric growth inhibition method was analyzed through Kruskal–Wallis tests and Dunn’s test for median comparison. Sponges with 2% chitosan were used as control samples for each analysis. All data were processed in a STATGRAPHICS PLUS 5.1 statistical package.

## 3. Results and Discussion

### 3.1. Physical Appearance Description of the Sponges

**Figure 1:**
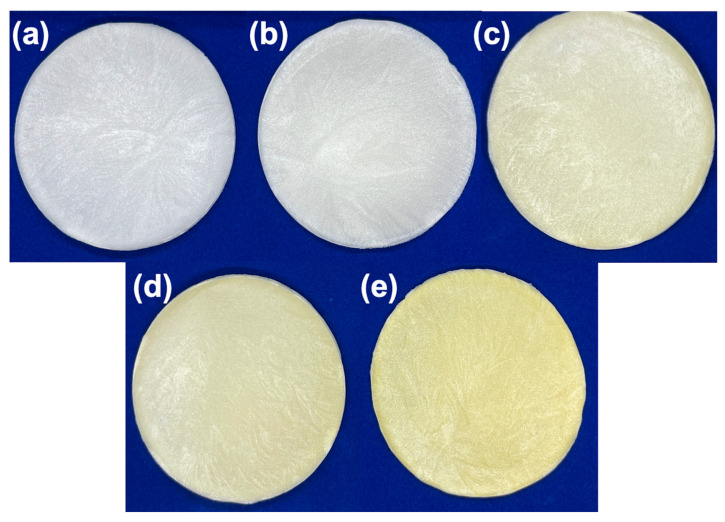
Physical appearance of chitosan–collagen–honey sponges: Ch 2% (**a**), ChC (**b**), ChC-20H (**c**), ChC-40H (**d**), and ChC-60H (**e**).

Freeze drying is commonly used to fabricate sponge-based biomaterials to produce three-dimensional systems with interconnected porous structures. The present work successfully produced chitosan–collagen–honey-based sponges as wound dressings (Figure 1).

The different treatments presented a porous, three-dimensional structure, each showing a soft, spongy, and slightly glossy surface. The Ch 2% and ChC sponges exhibited a smoother surface and appearance, while the honey-containing sponges had a slightly rougher structure. The treatments without honey exhibited a lighter color tone than the honey-containing composite formulations, which revealed a more yellow color and were brighter. In addition, the dimensions of the sponges were 80 mm in diameter, with varying thickness between treatments.

### 3.2. Physicochemical Characterization of Sponges

Color is an important property within the physical characterization of biomaterials since consumer acceptability will largely correspond to the visual aspect of the product [31]. All sponges exhibited an opaque three-dimensional structure with different optical characteristics between treatments. Table 2 shows the color parameters for each treatment. The Ch 2% and ChC sponges revealed a lighter coloration in L*, but no significant differences between both treatments (*p* < 0.05) were found. The incorporation of honey into the sponges resulted in a significant decrease (*p* < 0.05) in the L* values as the concentration increased, with the ChC-60H treatment exhibiting the lowest value for this parameter.

In the case of the a* and b* parameters, significant differences between treatments were observed (*p* < 0.05), and both variables showed an inverse behavior as described for parameter L* because the honey addition caused a gradual increase in the a* and b* values. The a* and b* ranged from −1.19 to 6.26 and 5.43 to 44.95, respectively. According to the CIE Lab scale, the addition of honey resulted in a slight increase in redness (a*) and a significant rise in yellowness in the sponges. Likewise, the Ch 2% and ChC treatments presented a lighter coloration, but the sponges with honey turned yellow with a darker appearance.

For Δ*E*, the addition effect of the compounds on the color change in the Ch 2% sponges was evaluated. A Δ*E* value below 5 is not considered a noticeable color change [32]. The ChC sponges did not undergo a perceptible color change when compared to Ch 2% sponges, which was barely perceptible (Δ*E* < 5). However, the addition of honey to the sponges made the treatments mostly perceptible. The Δ*E* revealed significant differences between treatments (*p* < 0.05), showing values ranging from 1.85 to 43.46 and increasing the color change concerning honey concentration in the sponges.

#### 3.2.1. FTIR Analysis

**Figure 2:**
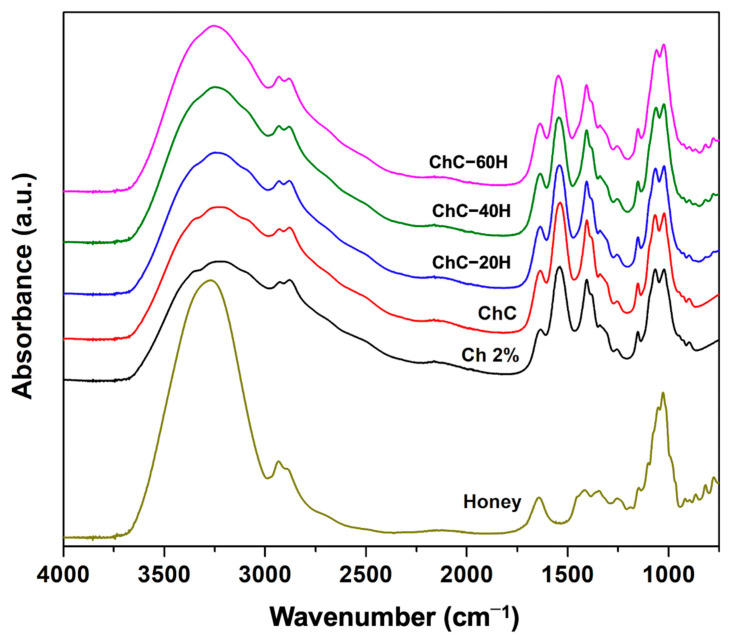
FTIR-ATR spectra of chitosan–collagen–honey-based sponges.

FTIR-ATR spectroscopy was used to identify functional groups and establish possible chemical interactions between sponge components [33]. The effect of the interactions among chitosan, collagen, and honey was analyzed through the changes in the FTIR spectra’s absorption bands. In this sense, Figure 2 shows the comparative study of the FTIR spectra obtained for each treatment in a wavenumber range of 4000–600 cm^−1^.

The characteristic spectra of Ch 2% sponges showed extensive absorption bands from 3348 to 3224 cm^−1^ and were associated with stretching vibrations of the O-H and N-H bonds of the primary amide [34]. The bands between 2925 and 2877 cm^−1^ corresponded to symmetric CH_2_ stretching vibrations, while the band at 1405 cm^−1^ was attributed to asymmetric CH_2_ bending vibrations of the pyranose ring [35,36]. In ChC sponges, the band at 1405 cm^−1^ was also attributed to the collagen’s O-H (COOH) group deformation. In Ch 2% sponges, the frequencies found at 1633 and 1538 cm^−1^ were attributed to N-H bond bending vibrations of the N-acetyl groups [23,37]. However, by incorporating collagen into sponges (ChC), the bands at 1633 cm^−1^ and 1538 cm^−1^ indicated C=O bond stretching (Amide I), and N-H and C-N bond vibrations (Amide II), respectively [38]. These absorption bands (Amides I and II), in conjunction with the peaks detected at 3348–3224 cm^−1^ suffered increases in the intensity of their signals, which could suggest possible interactions by hydrogen bonds among the C=O, O-H and N-H groups of chitosan and collagen. Previous studies have suggested these signals can be attributed to an ionic interaction between the protonated groups of chitosan (NH_2_^+^) and the anionic groups of collagen (COO^−^) [2]. Likewise, Ch 2% and ChC sponges revealed absorption bands at 1150 and 1020 cm^−1^ related to the C-O bond stretching vibrations of the polymeric chitosan structure. A small peak was identified at 895 cm^−1^ and was associated with stretching vibrations of the skeletal chitosan structure [39].

On the other hand, when the honey was incorporated into the ChC sponges, some changes in the signal intensity of the FTIR spectra were revealed. The analysis presented increases in the absorption band at 3348–3224 cm^−1^, showing overlaps of the O-H bonds over the N-H groups. In addition, a slight decrease was observed in the signal intensity of Amide I, corresponding to O-H groups bending (water molecules), C=O bond stretching vibrations of honey carbohydrates, and Amide I deformation [40]. Moreover, a slight decrease was noticed due to Amide I deformation, O-H group bending (water molecules), and C=O bond stretching vibration of honey carbohydrates [40]. Decreases in the Amide II band (N-H bending) and the band detected at 1405 cm^−1^ (O-H bending) were also identified. These modifications indicate possible intermolecular interactions by hydrogen bonds between the OH, NH_2_, and C=O groups of the sponge components [41].

Moreover, the intensity of the peaks from 1150 to 1020 cm^−1^ gradually increased due to the increase in C-C and C-O groups that conferred the honey addition. Moreover, the minor bands between 775 and 816 cm^−1^ confirmed the presence of honey in the sponges, and these bands were attributed to C-H groups bending vibrations and the carbohydrate ring that makes up honey [40,42].

#### 3.2.2. Morphological Characterization

**Figure 3:**
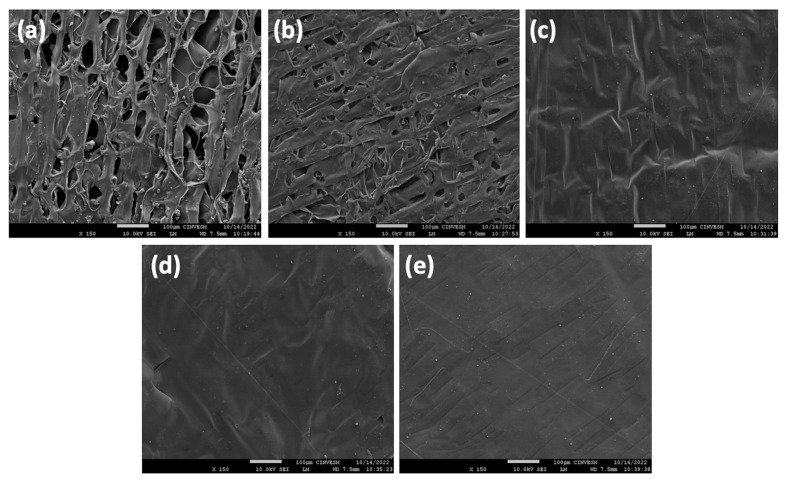
SEM surface micrographs of the chitosan–collagen–honey sponges: Ch 2% (**a**), ChC (**b**), ChC-20H (**c**), ChC-40H (**d**), and ChC-60H (**e**). The scale bar indicates 100 μm.

**Figure 4:**
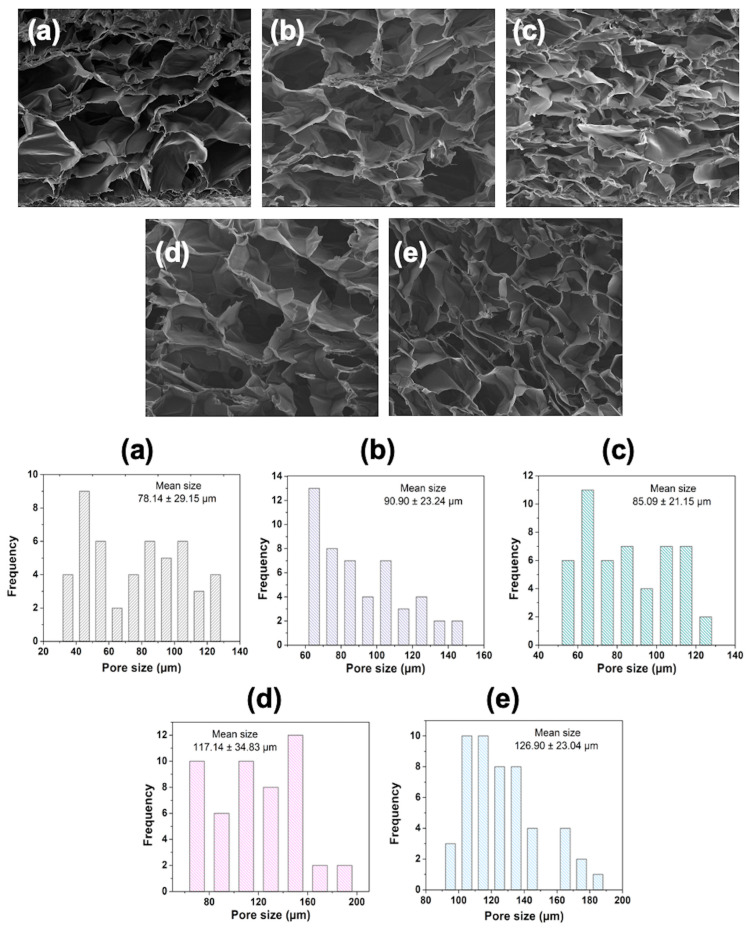
SEM cross-sectional micrographs and pore size distribution of chitosan–collagen–honey sponges: Ch 2% (**a**), ChC (**b**), ChC-20H (**c**), ChC-40H (**d**), and ChC-60H (**e**).

SEM analyzed the microstructure of chitosan-, collagen-, and honey-based sponges. For each treatment, micrographs were obtained from the surface (Figure 3) and cross-sectional (Figure 4) regions at magnifications of 150× and 200×, respectively. It was observed that the surfaces of all sponges had a uniformly dispersed and homogeneous distribution (Figure 3). In addition, the microstructure of the treatments revealed miscible blends with aggregates or phase separation absences, suggesting the components were well integrated into the biomaterial. This factor may be related to the formation of interactions among the elements, as has been indicated by the FTIR spectra. Ch 2% and ChC sponges presented porous structures with heterogeneous pore shapes and sizes. In contrast, the ChC-20H, ChC-40H, and ChC-60H exhibited a different morphological aspect because they lacked porosity and had striated and rough surfaces. Honey showed a negative effect on the pore formation in the surface region during the freeze-drying process. Escárcega-Galaz et al. reported similar behavior previously [18] when preparing chitosan films blended with honey. Other studies have suggested that incorporating plasticizing agents such as honey, glycerol, or sorbitol limits the formation of surface pores in chitosan matrices [43,44].

Figure 4 shows that the cross-sectional region of all treatments consisting of pores of irregular sizes and shapes. Nevertheless, despite the non-uniformity of the pores, there was no phase separation between the components, indicating the blends were homogeneous.

Figure 4 shows the pore size distribution determined by the cross-sectional area of each sponge treatment. It was observed that the honey composite sponges revealed a more defined unimodal distribution than those that did not contain honey, since the Ch 2% and ChC treatments showed a higher heterogeneity in their pore sizes. Nevertheless, none of the treatments revealed uniform unimodal distributions due to the presence of large or small pores, resulting in skewed distributions. The SEM results showed that the mean pore size of the sponges fluctuated from 78.14 to 126.90 µm, with the Ch 2% formulation having the smallest pore sizes, in contrast to ChC-60H, which revealed the largest pore sizes. The ChC sponges exhibited a mean pore size equivalent to 90.19 µm. Honey incorporation at 20% (Ch/Coll/17H) exhibited a mean pore size of 85.09 µm, which gradually increased to 117.14 µm and 126.90 µm with increasing honey concentration at 40% (ChC-40H) and 60% (ChC-60H), respectively. This effect can be attributed to the honey’s ability to incorporate into the polymeric chains and interact with them by hydrogen bonds, generating a larger free volume among the chains and consequently increasing the pore size [45].

#### 3.2.3. Porosity Degree

The porous structure and the porosity degree are the main properties that determine the absorption capacity of dressings [46] and provide wounds with a barrier permeable to oxygen and water but impermeable against microbial colonization. The porosity also provides suitable microenvironments to support cell proliferation, migration, and differentiation during tissue regeneration [47].

Table 3 shows the porosity degree analysis performed on sponges formulated with chitosan, collagen, and honey. The porosity percentage of the sponges ranged from 79.90 to 90.17%, with significant differences between treatments (*p* < 0.05). Ch 2% sponges presented the lowest porosity rates of all treatments. The addition of collagen and, subsequently, honey promoted considerable increases in the porosity degrees of the sponges. For these formulations, the porosity ranges fluctuated from 84.86 to 90.17, with the ChC and ChC-40H sponges having the highest porosity degrees, but no significant differences between both treatments were found (*p* < 0.05). Moreover, all the honey formulations showed higher porosities compared to the Ch 2% sponges. Sarhan et al. [48] reported similar trends in their porosity studies when developing chitosan/PVA/honey nanofibers. Likewise, Saberian et al. [49] presented porosities of 59.91%, 53.28%, and 60.21% in chitosan/alginate, chitosan/alginate/honey, and chitosan/alginate/honey/aloe vera sponges, respectively.

For cutaneous wound applications, the desirable porosity degree to facilitate exudate absorption in dressings should be 60–90% [50]. The sponges’ porosity in this study allows for sufficient use as wound dressings.

#### 3.2.4. Absorption Capacity

**Figure 5:**
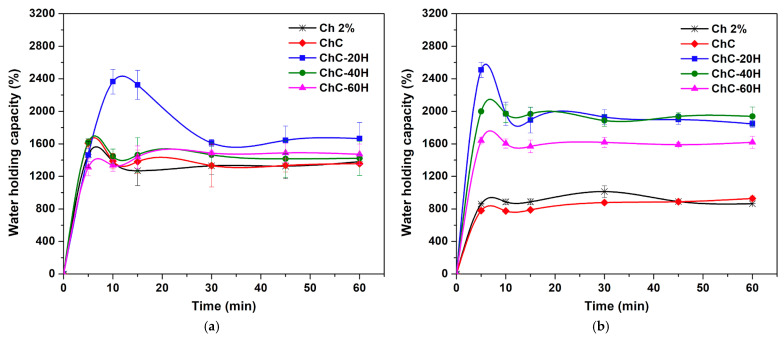
Absorption kinetics of chitosan–collagen–honey sponges in contact with deionized water (**a**) and PBS solution (**b**).

Figure 5 shows the absorption kinetics of the sponges evaluated in deionized water (Figure 5a) and PBS solution (Figure 5b) during 60 min. In biomedicine, the absorption capacity is a desirable factor in wound dressing because it allows the absorption of extracellular fluids and prevents their excessive accumulation in the wound bed [51,52]. In this work, all sponges reached absorption equilibrium after 30 min in deionized water and 15 min in PBS solution. At the end of the assay, the absorption rates in the sponges fluctuated in the range of 1357–1665% in deionized water, with the ChC-20H sponges having the highest absorption rates. Instead, it was revealed that the absorption rates ranged from 865 to 1938% in PBS solution, with higher values attributed to ChC-40H and ChC-60H sponges, and both treatments showed no significant difference in their absorption percentages.

In the assay’s first minutes, all treatments exhibited rapid initial absorption in both media absorption rates. However, when all treatments achieved equilibrium absorption, we observed a significant reduction in this property. This effect was mostly observed in honey composite sponges, which may be associated with the high honey solubility in water that facilitated the degradation of the dressing structure, as reported by Shamloo et al. [21]. This factor allowed honey composite sponges to reduce their absorption capacity until equilibrium. Specifically, ChC-20H sponges exhibited the maximum absorption rates in both hydrating agents, with values of 2364% at 10 min in contact with deionized water and 2510% at 5 min in contact with PBS. After these times, their absorption rates decreased considerably until reaching equilibrium.

On the other hand, the effect of honey promoted considerably increased absorption rates of sponges compared to the treatments without honey (Ch 2% and ChC). As previously described in this investigation, the pore size and the porosity degree primarily influence this characteristic. The presence of larger pores in these treatments may be the reason for the higher porosity degrees observed in honey composite sponges, as reported by several authors in the literature [44,48,49]. It explains the ability of the honey composite sponges (ChC-20H, ChC-40H, and ChC-60H) to hold higher rates of fluids (water and PBS) than the Ch 2% and ChC sponges.

Moreover, the sponges’ absorption rates differed in both hydrating mediums. For instance, Ch 2% and ChC sponges presented a higher adsorption capacity in deionized water compared to PBS solution. It is possibly due to the acidic pH of the deionized water, which supported the protonation of NH_2_ groups to NH_3_^+^, resulting in increases in positive charges and electrostatic repulsive forces [40,53]. In contrast, honey composite treatments revealed higher adsorption rates in PBS than in deionized water. This factor was attributed to the slightly basic pH of the PBS solution (pH = 7.4), which promoted the ionization of COOH groups to COO—groups, leading to increased electrostatic repulsion forces and consequently generating higher absorption rates [40].

#### 3.2.5. Barrier Properties

During the healing process, biomaterials must have the ability to control water losses in the wound and provide adequate moisture to prevent excessive dehydration and thus facilitate tissue healing [37]. For this purpose, Table 3 shows the water vapor permeability of each sponge formulation. This study revealed significant differences between treatments for each variable described (*p* < 0.05). The Ch 2% sponges exhibited the highest WVTR rates with 131.01 g h^−1^ m^−2^ values, while the lowest rates were attributed to the ChC-40H sponges (99.39 g h^−1^ m^−2^). These data suggest that Ch 2% sponges have the highest permeability due to free OH and NH_2_ groups that favor their affinity for water molecules [54].

On the other hand, it was observed that WVP was highest in ChC sponges, with values of 0.3255 g m^−1^ d^−1^ mm Hg^−1^, while the lowest WVP values were attributed to ChC-40H sponges, with rates of 0.2118 g m^−1^ d^−1^ mm Hg^−1^. When collagen was added to the sponges, more OH and NH_2_ functional groups were available, which promoted more significant interactions with water and contributed to the WPV increase in the ChC sponges compared to the Ch 2% sponges. The addition of honey also demonstrated a gradual decrease in the WVTR and WVP values. This behavior may be related to possible intermolecular interactions among the components of the treatments, which reduced the availability of free hydrophilic groups, resulting in a minor affinity for water. Saberian et al. [49] demonstrated a similar trend when they prepared chitosan/alginate/honey-based hydrogels. Their study reported a WVTR of 33 g h^−1^ m^−2^ in chitosan/alginate composite hydrogels. However, adding honey to their membranes significantly decreased 16 g h^−1^ m^−2^ values. Singh et al. [54] also reported this effect by developing cross-linked membranes composed of dextran/nano-soy/glycerol/chitosan/honey.

According to Andonegi et al. [2], healthy skin can reach WVTR values of 8.5 g h^−1^ m^−2^. However, these values can reach 12 g h^−1^ m^−2^ in wounds and 214 g h^−1^ m^−2^ for burns. This study indicates that wound dressings must possess these values to promote optimal gas exchange, which supports the healing process. Our treatments fall within permeability ranges for skin wound applications in this case. Similarly, Ch 2% and ChC sponges exhibited the highest WVTR and WVP rates, respectively, making them the most effective treatments for preventing dehydration and maintaining moist healing environments.

#### 3.2.6. Mechanical Properties

The mechanical properties of the sponges (thickness, tensile strength, elasticity modulus, and elongation at break) are shown in Table 4. The mechanical properties represent one of the most important requirements for wound dressings, since they must withstand continuous tensile loads and not suffer fractures or tears during their handling [9,55]. In this work, the stress–strain curves were used to analyze the various mechanical parameters of the sponges. As it is possible to observe, some significant differences among the treatments were revealed for each mechanical parameter described (*p* ˂ 0.05).

Ch 2% sponges showed the lowest thickness of all treatments (1.07 ± 0.06 mm), while the collagen and honey incorporation generated considerable increases in the thickness of the sponges, with the ChC-40H formulation having the highest thickness of all treatments (1.96 ± 0.06 mm). The thickness could have influenced the barrier properties of the sponges (WVTR and WVP), as it was revealed that higher thicknesses resulted in lower permeabilities in the sponges. In this case, honey-loaded sponges revealed lower permeability rates compared to sponges that did not contain honey. Lemus et al. [56] also found that adding melipona honey to graphene-agar oxide film made them thicker but considerably decreased the WVTR and WVP values of the films.

On the other hand, the ranges for tensile strength were 0.377–0.461 MPa, while the elongation at break ranged from 7.56 to 16.36%, with the Ch 2% sponges exhibiting the highest values in both properties. It was found that honey incorporation into sponges caused a decrease in the parameters of tensile strength and elongation rates, with the lowest values corresponding to ChC-20H sponges in both properties. The reduction in the tensile strength values could indicate a possible plasticizing behavior of honey in the treatments [40,56].

The Ch 2% and ChC treatments had the strongest tensile strengths because the materials in the sponges show strong chemical interactions among them, especially since the chitosan structure produces this behavior. However, several studies have reported that honey can enter the polymeric chains of chitosan and disrupt their intermolecular interactions. This mechanism reduces the friction between the chain and rigidity, generating a greater free volume that facilitates chain mobility [18,45]. Koosha et al. [57] prepared chitosan/PVA composite hydrogels in blends with allantoin and bee honey to report on the plasticizing effect of honey. Soininen et al. [58] noted that the presence of monosaccharides like fructose and glucose primarily contributes to the plasticizing effect of honey. They reported on this mechanism by plasticizing whey protein films with acacia honey.

On the other hand, the elasticity modulus of the treatments ranged from 7.95 to 12.49 MPa; the formulation ChC-20H showed the highest modulus, and the ChC sponges had the lowest modulus. These results indicate a considerable reduction in ChC sponges compared to the treatment added with Ch 2%. However, the ChC-20H treatment saw an increase in the modulus, while the ChC-40H and ChC-60H treatments showed a gradual decrease. This behavior confirmed the plasticization effect of honey in the treatments, as previously mentioned in the tensile strength parameter. Moreover, the elongation rates of the sponges were negatively affected when honey was incorporated into the treatments, especially in ChC-20H sponges, which showed the lowest elongation rates (7.56%). Nevertheless, an increase in this property was again observed in the ChC-40H and ChC-60H treatments with values of 9.65% and 8.39%, respectively, but the elongation was still considerably lower than the Ch 2% and ChC sponges.

The Ch 2% and ChC treatments exhibited higher stiffness according to the tensile strength and modulus parameters. Still, they resulted in more elastic structures than the honey-loaded sponges, as indicated by the elongation parameter. It demonstrated that the honey composite sponges exhibited higher flexibility but lower elongation. The above suggests that the addition of honey considerably reduced the mechanical properties of the dressings despite providing plasticization to them. However, the microstructural morphology changes in honey-incorporating treatments significantly influence this behavior. The above theory could be supported by Adhikari et al. [44], who suggested that by incorporating honey into polycaprolactone composite nanofibers, the pore size and porosity degree increased for honey concentration, leading to reduced mechanical properties (tensile strength and modulus) of the dressings. Rajput et al. [59] also confirmed this behavior by incorporating honey into three-dimensional silk fibroin porous scaffolds. In the present investigation, the honey-loaded sponges exhibited a higher degree of porosity because they showed a bigger pore size, according to the micrographs obtained by SEM. Adhikari et al. reported a lower mechanical resistance to honey-loaded sponges, which could explain this effect. Furthermore, the polydispersity in the pore size of the sponges is another factor that could support this mechanical behavior.

However, it has been reported that wound dressings should provide mechanical properties and flexibilities analogous to or even greater than skin, which comprises tensile strength ranges of 0.1–32 MPa and elongation rates of 0.42–2.26% [42]. In our case, the chitosan–collagen–honey sponges provide adequate mechanical properties for wound application.

#### 3.2.7. TGA

**Figure 6:**
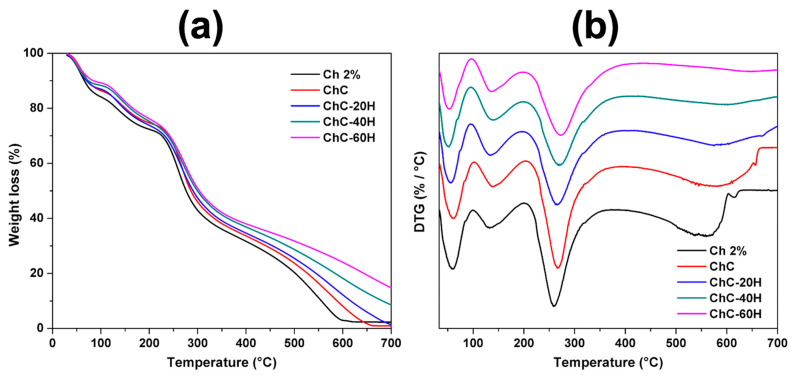
TGA (**a**) and DTGA (**b**) thermograms of chitosan–collagen–honey sponges.

The TGA studies evaluated the thermal degradation of the sponges by measuring weight loss as a function of increasing temperature. In the present work, it should be considered that the sponges maintain adequate thermal stability since their physicochemical and biological functions may be limited if their components are degraded during their application to skin wounds. The comparative TGA thermograms and DTGA curves of the sponges are shown in Figure 6.

In all treatments, thermograms exhibited four successive weight losses (Figure 6a). The first weight loss fluctuated between 30 and 100 °C and generated 10 to 15% weight loss. The second loss occurred from 100 to 200 °C, and it suffered weight loss from 11 to 14%. The third loss was shown at temperatures of 200–370 °C, resulting in 36–39% weight loss, and the fourth loss happened at temperatures of 370–670 °C, resulting in 23–35% weight loss. The residue generated at the end of the run was considered ash. Within the temperature ranges described, the higher accumulative weight loss was found in Ch 2% sponges compared to the ChC-40H sponges with the lowest weight loss.

The DTGA curves of chitosan–collagen–honey sponges are presented in Figure 6b. The peaks found in the curves were associated with the thermal decomposition stages of the different sponge components. The DTGA analysis revealed four decomposition stages for each treatment, and the maximum decomposition temperatures (*Td_max_*) for each stage are listed in Table S1. The first decomposition stage was due to the water evaporation absorbed from the sponges (30–100 °C) [45]. For this stage, *Td_max_* ranged from 51 to 61 °C, with ChC sponges having the highest *Td_max_*.

The second decomposition stage was found in *Td_max_* ranges of 132–139 °C. It was associated with water loss strongly linked to the structure of the polymers and the loss of other volatile compounds [60]. In this section, the *Td_max_* ranges were from 132 to 139 °C, assigning the highest *Td_max_* values to ChC-40H sponges.

The third decomposition stage corresponded to ether bond breakage and NH_2_ and CH_2_OH chitosan group degradation [60,61]. In addition, during this temperature interval, collagen thermal degradation occurred alongside the decomposition and carbonization of honey carbohydrates [2,48]. At this stage, *Td_max_* ranges from 258 to 273 °C were observed, attributing a gradual increase in *Td_max_* as the honey concentration in the treatments increased.

The largest *Td_max_* fluctuations happened in the fourth decomposition stage, where 560–646 °C temperature ranges were revealed. The pyranose ring breakage of the chitosan and the organic matter oxidation of honey correlated with this stage [48]. The increase in honey concentration resulted in considerable increases in ash residues at the end of the run. Radoor et al. [45] reported the same trend when preparing PVA/chitosan films blended with different honey concentrations.

#### 3.2.8. DSC Analysis

**Figure 7:**
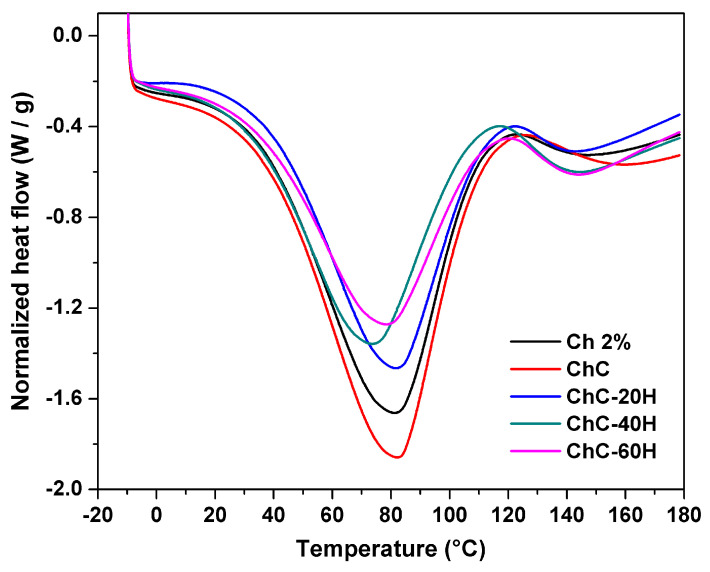
Comparative DSC thermogram of chitosan–collagen–honey sponges.

The DSC thermograms of the sponges are shown in Figure 7. All treatments under heating at temperatures from −10 to 180 °C revealed the existence of two endothermic peaks associated with different transition phases. Table S1 compiles the data corresponding to the transition parameters obtained from the DSC thermograms.

An extensive endothermic peak located between 20 and 120 °C was attributed to the removal of water and residual acetic acid that was bound in the polymeric chains of the sponges [57]. The Ch 2%, ChC, and ChC-20H treatments did not show considerable changes in their maximum dehydration temperatures (*T_d_*), since they showed values of 80.76 °C, 81.73 °C, and 81.20 °C, respectively. Nevertheless, slight decreases in *T_d_* were observed in ChC-40H and ChC-60H sponges, with values of 73.25 °C and 77.76 °C, respectively. Moreover, to perform this transition, Ch 2% and ChC sponges had the highest endothermic enthalpies (Δ*H_d_*) values of 351.70 and 402.76 J/g, respectively. This result indicates that Ch 2% and ChC sponges required higher temperatures and energy to evaporate water molecules due to the interaction of these treatments. In this sense, Meng et al. [62] reported the formulation of chitosan/alginate/hyaluronic acid composite sponges cross-linked with genipin. Moreover, Singh et al. [54] suggested this behavior can be attributed to free OH and NH_2_ groups found in chitosan and collagen molecules, which may enhance the affinity for water molecules in Ch 2% and ChC sponges. However, the affinity for water molecules decreased in honey-loaded sponges due to possible interactions formed (hydrogen bonds) between the components, as previously described in the FTIR spectra (Figure 2). This mechanism caused a reduction in the enthalpies of the honey-loaded treatments.

A second endothermic peak was found between 120 and 180 °C and corresponded to the decomposition temperature of the polymers (*T_d_*_2_). The Ch 2% sponges showed a *T_d_*_2_ of 147 °C, and the enthalpy to carry out this transition (Δ*H_d_*_2_) was 16.73 J/g. The *T_d_*_2_ increased in the ChC treatment, presenting *T_d_*_2_ values of 155 °C. Incorporating honey into the sponges resulted in significant decreases in *T_d_*_2_, while the honey-loaded treatments showed no significant changes in *T_d_*_2_. This effect may be due to the plasticizing behavior conferred by honey, which has been widely discussed in the literature [18,56]. Conversely, we observed that the addition of honey to the treatments led to an increase in Δ*H_d_*_2_ values when compared to sponges without honey. Such behavior was associated with structural water loss as well as the breaking of the polymeric chains’ bonds [60].

### 3.3. Antibacterial Activity

**Figure 8:**
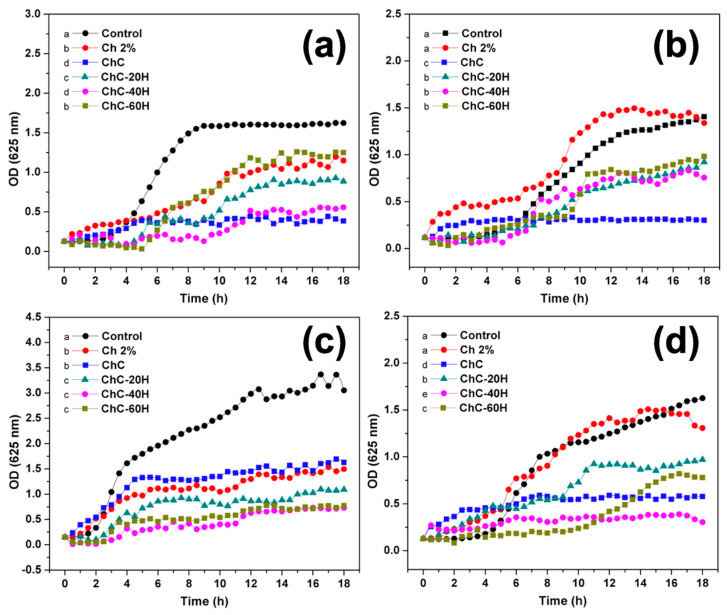
Effect of chitosan–collagen–honey sponges on the bacterial growth curve of *Staphylococcus aureus* (**a**), *Pseudomonas aeruginosa* (**b**), *Escherichia coli* (**c**), and *Listeria monocytogenes* (**d**). Different literals represent significant differences according to Dunn’s test (*p* < 0.05).

The antibacterial activity of the sponges against *S. aureus*, *P. aeruginosa*, *L. monocytogenes*, and *E. coli* was evaluated through the inhibition growth curves (Figure 8). This study examined four pathogenic bacteria, known to be highly prevalent in skin ulcers [6,63].

When evaluating the bacterial activity of the sponges against *S. aureus* and *P. aeruginosa*, it was observed that the ChC formulation had the highest inhibitory activity of all treatments. However, ChC did not show a significant difference (*p* < 0.05) for ChC-40 h sponge when evaluated against *S. aureus.* For *L. monocytogenes*, ChC-40 h sponges presented the lowest O.D., suggesting this treatment showed the highest inhibitory effect on the bacteria. In the *E. coli* curve, the honey-loaded treatments did not present significant differences (*p* < 0.05) among them. They showed the lowest optical densities, indicating that only *E. coli* showed a greater antibacterial effect than the sponges loaded without honey (Ch 2% and ChC).

On the other hand, compared with the Ch 2% treatment, it was well observed that the honey-loaded treatments exhibited significant differences (*p* < 0.05) and greater inhibitory capacity against *P. aeruginosa*, *E. coli*, and *L. monocytogenes*. The *S. aureus* kinetics showed that the ChC-60H treatment did not present statistically significant differences compared to Ch 2% (*p* < 0.05).

Currently, the antibacterial mechanism of chitosan has not yet been fully defined. Previous studies have reported that the antibacterial capacity of chitosan is mainly attributed to the presence of cationic amino groups (NH_3_^+^) in its structure, which can interact with the cell wall of bacteria that are composed of negatively charged components (COO-) like peptidoglycans and teichoic acids in Gram-positive (+) bacteria or components such as lipopolysaccharides and phospholipids in Gram-negative (−) bacteria. Through these interactions, chitosan can form strong electrostatic interactions with the cell wall and cause its breakage, leading to the leakage of intracellular components and osmotic imbalances, resulting in cell death [21,64]. It has also been reported that chitosan in Gram-negative (−) bacteria can hydrolyze the cell wall, acting as a barrier that prevents the entry of nutrients into the cell [45].

In addition, it has been reported that the antibacterial capacity of honey is due to its high acidity, the constant production of hydrogen peroxides, the presence of flavonoids, and its high osmotic effect attributed to the supersaturated concentration of sugars [2,65]. Another mechanism proposed by Mukhopadhyay et al. [66] suggests that hypochlorous acids (by-products obtained through hydrogen peroxide degradation by the action of myeloperoxidases present in honey) may provide antibacterial responses by interposing themselves during base pairing in the bacterial DNA.

In the present study, it is possible to observe differences in the antibacterial activity of the treatments. The addition of collagen into the sponges (ChC) significantly improved the antibacterial activity compared to Ch 2% sponges. However, the addition of honey in the treatments resulted in different trends and behaviors in the antibacterial activity of the treatment used. Likewise, it is observed that in some cases (*S. aureus* and *P. aeruginosa*), the addition of honey did not exhibit improvements in antibacterial efficiency in the treatments as opposed to the sponges without honey. This factor may be strongly influenced by the molecular interactions that occurred between the components of the sponges, which largely modified the antibacterial properties of the treatments. In general, chitosan and collagen present amino groups (NH_2_) that confer cationic nature and antibacterial properties. However, the incorporation of honey affects the molecular structure of both polymers by interacting with them through hydrogen bonds, which can reduce the availability of free amino groups and thus the antibacterial efficiency of the treatments in some cases [39]. However, there are other factors in honey, such as its high osmolarity, its acidic nature, and the presence of hydrogen peroxides, flavonoids, and phenolic acids, which contribute to its antibacterial efficiency [40]. However, there are other factors of honey, such as its high osmolarity, its acidic nature, the presence of hydrogen peroxides, flavonoids, and phenolic acids, which contributed to its antibacterial efficiency in the treatments [21,22]. Therefore, it was shown honey components in the treatments were more effective against *E. coli* and *L. monocytogenes* compared to *S. aureus* and *P. aeruginosa*.

### 3.4. Cell Viability and Cytotoxicity

**Figure 9:**
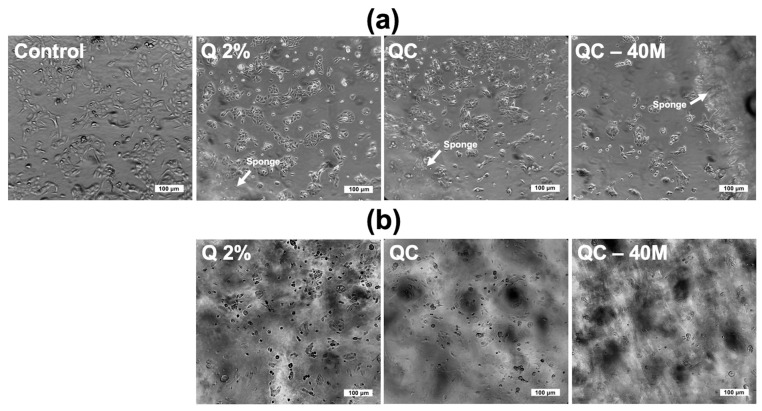
Effect of the chitosan–collagen–honey sponges on the viability and growth cell of HaCaT in the peripheral (**a**) and inner (**b**) regions of the treatments.

Figure 9 illustrate the viability of chitosan–collagen–honey sponges on HaCaT cells. We selected Ch 2%, ChC, and ChC-40H sponges because they had the best cell viability results according to preliminary studies and presented the best antibacterial properties.

Studies with HaCaT cells indicated that Ch 2% and ChC sponges exhibited cell growth comparable to the control in the peripheral region of the treatments (Figure 9a). However, cell proliferation was significantly reduced in ChC-40H sponges. Furthermore, in the inner area of the treatments (Figure 9b), it was observed that the highest cell growth was found in Ch 2% sponges, whereas in ChC-40H, there was low HaCaT proliferation. However, none of the three treatments revealed the presence of damaged or destroyed cells, indicating that the sponges were not cytotoxic to HaCaTs. The Ch 2% treatments promoted the highest cell proliferation and were the most biocompatible with HaCaT.

It was revealed that the Ch 2% and ChC sponges showed higher proliferation, and growth of HaCaT cells, with a greater effect in Ch 2% sponges. On the other hand, adding honey to the sponges (ChC-40H) negatively affected the viability of HaCaT. The literature has extensively discussed how porosity characteristics, particularly pore size or degree, and intermolecular interactions among dressing components can affect cell viability; nonetheless, the available evidence does not define whether the ChC-40H treatment presented a mechanism of cytotoxicity in the cells.

Noori et al. [40] reported similar results to this study when showing that the viability of human fibroblasts was higher in chitosan 2% (*w*/*v*) hydrogels compared to hydrogels composed of chitosan/PVA/honey/nano-clay. This author mentioned that this behavior can be attributed to free amino groups (NH_2_) in chitosan, which can generate cell attraction (fibroblast or keratinocytes) because the cell membrane is composed of negatively charged groups, promoting more outstanding cell adhesion and proliferation. However, when the amino groups of chitosan interact with other functional groups, it is evident that the availability of these groups decreases and promotes less cell adhesion and proliferation. It could prove accurate since, in this study, chitosan can interact via hydrogen bridges with functional groups of honey and collagen through hydrogen bonding, as demonstrated in the FTIR spectroscopy studies. These interactions can lead to a decrease in the availability of free amino groups, which may harm the viability of HaCaT cells.

Similarly, other researchers have indicated that the pore size of biomaterials can significantly affect cell viability. Reports suggest that an adequate pore size can enhance cell interconnection. However, lacking adequate size, the cells will have no reason to settle between the cavities and promote adhesion and proliferation [49]. Likewise, large pore sizes may reduce cell interconnections and cell proliferation. Zarei et al. [67] reported a similar characteristic in electrospun nanofibers based on polypyrrole/chitosan/collagen. In this case, the ChC-40H sponges had the largest pore size, which could result in a less suitable size for HaCaT growth than the Ch 2% and ChC sponges.

#### In Vitro Cellular Healing and Cytotoxicity Tests

**Figure 10:**
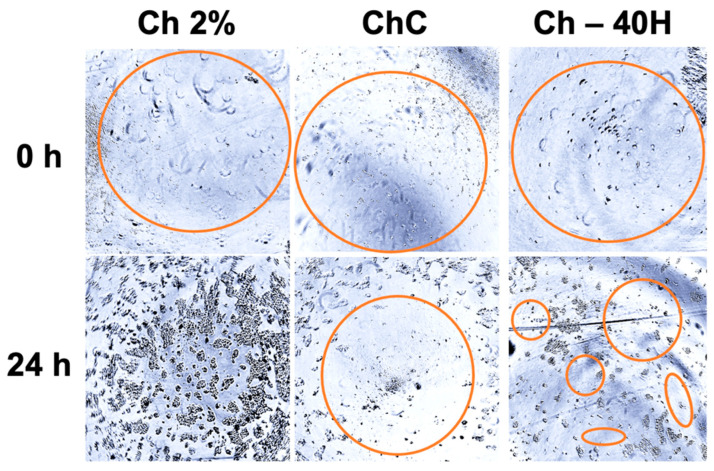
Effect of chitosan–collagen–honey-based sponges on in vitro cell healing with HaCaTs.

The effect of the sponges on in vitro cellular healing using HaCaTs is presented in Figure 10. The test indicated a shorter duration (24 h) of in vitro wound healing. In this study, the Ch 2% sponges had largely filled the wound area, resulting in the treatment that achieved the highest rate of wound compaction. However, we observed empty spaces where HaCaTs were no longer able to proliferate beyond 24 h. The ChC and ChC-40H sponges were also carrying out healing, but with more delayed and different effects. It was observed that ChC sponges were filling the wound area from the edges, while in ChC-40H sponges, the cells first migrated to the center of the wound area and performed their proliferation actions. The proliferation and migration rates in both treatments were much lower than those found in Ch 2% sponges. Furthermore, the three treatments did not show the presence of any destroyed or malformed cells. In this model, incorporating honey into treatments resulted in a more prolonged mechanism for wound compaction. The factors described above (porosity characteristics and intermolecular interactions) in the cell viability studies may be responsible for this delayed healing effect.

## 4. Conclusions

Polymeric biomaterials can effectively incorporate honey as a compound. In the present investigation, we successfully fabricated sponges formulated with chitosan, collagen, and honey. The treatments had flexible structures with a shiny, soft, and spongy appearance. The formation of porous structures during freeze drying provided permeability and high absorption rates to the sponges, especially in honey-loaded systems. These properties are relevant in biomedicine because they promote gas exchange and exudate absorption during healing. Moreover, mechanical properties showed that the different sponges were resistant to tensile stimuli and exhibited elastic and flexible properties. To evaluate the thermal degradation of the dressings, thermograms obtained by TGA and DSC indicated that all treatments showed excellent thermal stability. Furthermore, in vitro tests indicated that chitosan–collagen–honey dressings possess suitable antibacterial activity against *S. aureus*, *P. aeruginosa*, *E. coli*, and *L. monocytogenes*. The studies on antibacterial activity and preliminary cell viability tests led to the selection of the Ch 2%, ChC, and ChC-40H treatments for in vitro cell viability and healing tests against HaCaT cells. None of the treatments presented cellular cytotoxicity and promoted wound compaction. Ch 2% sponges provided the most favorable effects, while ChC-40H had reduced effects on viability and wound healing. The favorable physicochemical, antibacterial, and biocompatibility properties of chitosan/collagen/honey sponges highlight their potential as a promising alternative for clinical use in skin wound management.

## Figures and Tables

**Table 1:** Composition of the formulated chitosan–collagen–honey sponges.

Sponge Treatment	Ch (g)	Coll (g)	H (g)
Ch 2%	2.0	0	0
ChC	2.0	0.25	0
ChC-20H	2.0	0.25	0.45
ChC-40H	2.0	0.25	0.90
ChC-60H	2.0	0.25	1.35

Ch: chitosan, Coll: collagen, H: honey. Solute (g/100 mL).

**Table 2:** Chromatic parameter quantification of chitosan–collagen–honey sponges.

Treatment	L*	a*	b*	Δ*E*
Ch 2%	89.04 ± 0.74 ^d^	−1.19 ± 0.08 ^c^	5.43 ± 0.09 ^a^	NQ
ChC	88.23 ± 0.14 ^d^	−1.56 ± 0.06 ^b^	7.02 ± 0.14 ^b^	1.85 ± 0.12 ^a^
ChC-20H	87.16 ± 0.65 ^c^	−4.45 ± 0.11 ^a^	27.00 ± 0.73 ^c^	22.23 ± 0.60 ^b^
ChC-40H	78.10 ± 0.40 ^b^	4.50 ± 0.48 ^d^	42.95 ± 0.42 ^d^	39.66 ± 0.49 ^c^
ChC-60H	72.42 ± 0.40 ^a^	6.26 ± 0.25 ^e^	44.95 ± 0.45 ^e^	43.46 ± 0.45 ^c^

The data was reported according to the mean value ± standard deviation of nine measurements. Different literals in the same column represent significant differences (*p* < 0.05). NQ = not quantified.

**Table 3:** Porosity degree and barrier properties of chitosan–collagen–honey sponges.

Treatment	Porosity (%)	WVTR (g h^−1^ m^−2^)	WVP (g m^−1^ d^−1^ mm Hg^−1^)
Ch 2%	79.90 ± 1.60 ^a^	131.01 ± 3.69 ^d^	0.2456 ± 0.0328 ^ab^
ChC	90.17 ± 2.48 ^c^	122.34 ± 2.63 ^c^	0.3255 ± 0.0144 ^c^
ChC-20H	81.23 ± 0.94 ^b^	107.62 ± 6.89 ^b^	0.2475 ± 0.0098 ^b^
ChC-40H	90.13 ± 0.43 ^c^	99.39 ± 4.68 ^a^	0.2118 ± 0.0257 ^a^
ChC-60H	84.17 ± 1.25 ^b^	100.91 ± 3.00 ^ab^	0.2321 ± 0.0185 ^ab^

The data were reported according to the mean value ± standard deviation of three measurements. Different literals in the same column represent significant differences (*p* < 0.05).

**Table 4:** Mechanical properties of chitosan–collagen–honey sponges.

Treatment	Thickness (mm)	Tensile Strength (MPa)	Elasticity Modulus (MPa)	Elongation at Break (%)
Ch 2%	1.07 ± 0.06 ^a^	0.461 ± 0.044 ^c^	11.30 ± 2.30 ^c^	16.36 ± 0.37 ^d^
ChC	1.80 ± 0.05 ^b^	0.435 ± 0.036 ^bc^	7.95 ± 0.71 ^a^	13.71 ± 0.86 ^c^
ChC-20H	1.91 ± 0.04 ^cd^	0.377 ± 0.047 ^a^	12.49 ± 2.12 ^c^	7.56 ± 1.64 ^a^
ChC-40H	1.96 ± 0.06 ^d^	0.403 ± 0.069 ^ab^	10.95 ± 1.33 ^bc^	9.64 ± 0.61 ^b^
ChC-60H	1.89 ± 0.03 ^c^	0.394 ± 0.017 ^ab^	9.29 ± 0.86 ^ab^	8.39 ± 0.85 ^a^

The data were reported according to the mean value ± standard deviation of six measurements. Different literals in the same column represent significant differences (*p* < 0.05).

## Data Availability

All data and contributions are available in this article. For further information, the corresponding author can be contacted.

## Supplementary Materials

The following supporting information can be downloaded at: https://www.mdpi.com/article/10.3390/polym17172379/s1, Table S1: Thermal characterization data of sponges based on chitosan-collagen-honey.
