# Supplementary material for: Bioengineered Chitosan–Collagen–Honey Sponges: Physicochemical, Antibacterial, and In Vitro Healing Properties for Enhanced Wound Healing and Infection Control
Source: Polymers (Basel). 2025 Aug 31;17(17):2379. doi: 10.3390/polym17172379 (PMC12431070; doi:10.3390/polym17172379)
Supplement: Supplementary file 1 [file polymers-17-02379-s001.zip › polymers-3736066-supplementary.pdf]

Supplementary Materials

Table S1. Thermal characterization data of sponges based on chitosan-collagen-honey.

Treatment	TGA/DTGA parameters				Endothermic transition parameters DSC			
	$T_{d\ max1}$ (ºC)	$T_{d\ max2}$ (ºC)	$T_{d\ max3}$ (ºC)	$T_{d\ max4}$ (ºC)	T_{d1} (ºC)	ΔH_{d1} (J/g)	T_{d2} (ºC)	ΔH_{d2} (J/g)
Ch 2%	58.02	132.14	258.15	560.12	80.76	351.70	147.39	16.73
ChC	61.46	138.78	266.96	578.33	81.73	402.76	154.88	13.42
ChC-20H	55.94	134.63	266.55	573.74	81.20	300.92	144.55	24.56
ChC-40H	51.48	139.27	269.18	601.80	73.25	272.92	143.84	35.77
ChC-60H	53.25	137.70	272.60	646.39	77.76	249.52	144.48	32.39

$T_{d\ max1}$ = Maximum decomposition temperature of stage 1; $T_{d\ max2}$ = Maximum decomposition temperature of stage 2; $T_{d\ max3}$ = Maximum decomposition temperature of stage 3; $T_{d\ max4}$ = Maximum decomposition temperature of stage 4; T_{d1} = Dehydration temperature of stage 1; T_{d2} = Dehydration temperature of stage 2; ΔH_{d1} = Dehydration enthalpie of stage 1; ΔH_{d2} = Dehydration enthalpie of stage 2.
